# Cerebral Activations Related to Writing and Drawing with Each Hand

**DOI:** 10.1371/journal.pone.0126723

**Published:** 2015-05-08

**Authors:** Adriaan R. E. Potgieser, Anouk van der Hoorn, Bauke M. de Jong

**Affiliations:** 1 Department of Neurology, University Medical Center Groningen, University of Groningen, Groningen, The Netherlands; 2 Neuroimaging center, University Medical Center, University of Groningen, Groningen, The Netherlands; 3 Department of Neurosurgery, University Medical Center Groningen, University of Groningen, Groningen, The Netherlands; 4 Department of Radiology, University Medical Center Groningen, University of Groningen, Groningen, The Netherlands; University Of Cambridge, UNITED KINGDOM

## Abstract

**Background:**

Writing is a sequential motor action based on sensorimotor integration in visuospatial and linguistic functional domains. To test the hypothesis of lateralized circuitry concerning spatial and language components involved in such action, we employed an fMRI paradigm including writing and drawing with each hand. In this way, writing-related contributions of dorsal and ventral premotor regions in each hemisphere were assessed, together with effects in wider distributed circuitry. Given a right-hemisphere dominance for spatial action, right dorsal premotor cortex dominance was expected in left-hand writing while dominance of the left ventral premotor cortex was expected during right-hand writing.

**Methods:**

Sixteen healthy right-handed subjects were scanned during audition-guided writing of short sentences and simple figure drawing without visual feedback. Tapping with a pencil served as a basic control task for the two higher-order motor conditions. Activation differences were assessed with Statistical Parametric Mapping (SPM).

**Results:**

Writing and drawing showed parietal-premotor and posterior inferior temporal activations in both hemispheres when compared to tapping. Drawing activations were rather symmetrical for each hand. Activations in left- and right-hand writing were left-hemisphere dominant, while right dorsal premotor activation only occurred in left-hand writing, supporting a spatial motor contribution of particularly the right hemisphere. Writing contrasted to drawing revealed left-sided activations in the dorsal and ventral premotor cortex, Broca’s area, pre-Supplementary Motor Area and posterior middle and inferior temporal gyri, without parietal activation.

**Discussion:**

The audition-driven postero-inferior temporal activations indicated retrieval of virtual visual form characteristics in writing and drawing, with additional activation concerning word form in the left hemisphere. Similar parietal processing in writing and drawing pointed at a common mechanism by which such visually formatted information is used for subsequent sensorimotor integration along a dorsal visuomotor pathway. In this, the left posterior middle temporal gyrus subserves phonological-orthographical conversion, dissociating dorsal parietal-premotor circuitry from perisylvian circuitry including Broca's area.

## Introduction

Writing is a complex manual motor skill, gradually acquired during childhood [1]. In general, motor skills of the upper extremities are characterized by a combination of fine distal finger and hand movements and more proximal, spatial arm movements. Regarding the underlying cerebral organization of such movements, the primary motor cortex (M1) provides the main output to the spinal cord level that controls the upper limbs, although the ventral premotor cortex (PMv) makes a small contribution too, particularly to the segments that control distal hand movements [2,3]. The main output of the PMv, however, goes to the hand presentation of the primary motor cortex [4]. The dorsal premotor cortex (PMd) has a stronger role in proximal arm movements to navigate the arm in surrounding space to a target location [5]. This differential contribution of the PMv and PMd to distal and proximal movements is consistent with the somatotopy of the primary motor cortex and the relative positions of the PMv and PMd rostrally to it [6]. Likewise, such somatotopic relationship within the premotor cortex is also expressed in the distribution of responses in the PMv and PMd evoked by visually observed distal and proximal body movements, respectively [7,8].

The apparent role of the premotor cortex in supporting M1 includes its contribution to sensorimotor transformations required for goal-directed movements [9–14]. Such a critical role in the execution of complex movement sequences has been acknowledged for many decades [15]. In this respect, writing is similarly constituted by complex movement sequences while it requires additional integration of linguistic functions. It thus seems plausible that part of particularly the left (ventral) premotor cortex plays a role in the integration of motor and language functions. The left PMv is located adjacent to Broca’s area and these areas share cytoarchitectural characteristics [16,17]. This is further reflected by a distal movement dominance in right-hand writing, which is not the case when right-handed subjects write with their left hand [18,19]. The latter suggests that such an intimate relationship between the PMv and frontal language circuitry is lacking in the non-dominant hemisphere. While the left hemisphere is dominant for language [20], right-hemisphere dominance is particularly described for spatial processing including aspects of visuomotor integration [21–26].

In the present study, functional magnetic resonance imaging (fMRI) was used in right-handed subjects to identify cerebral activations related to right- and left-hand writing. By comparing these conditions we aimed to test the hypothesis that writing is based on general hemisphere-specific motor functions discerning fine precision and spatial movements related with the left and right hemisphere, respectively, reflected by a differential involvement of the left and right PMv and PMd.

Considering the intrinsic complexity of manual writing movements, the suggestion arises that writing includes a component of complex tool use, although writing can be effectively performed with paint on a single fingertip. Similar arguments hold for drawing. This generates the question to what extent writing is essentially an expression of general motor skill, indeed connected with language functions, or whether writing emerges from a unique location or circuitry. To effectively use a pencil, spinal cord efferents of the primary motor cortex directly control the motor units enabling independent (contralateral) finger movements [2,27], while the primary motor cortex receives information concerning sensorimotor transformations from the premotor cortex to guide goal-directed performance implicated in such writing. The convergence of sensorimotor information within extended cerebral circuitry to premotor regions places the latter in a logical position to similarly mediate language-motor transformations.

Historically, writing has been attributed to a specific brain region. Exner was the first to describe the neurological condition of isolated or pure agraphia, caused by a lesion in the posterior part of the left middle frontal gyrus (mFG) [28], at a location later functionally coined as premotor cortex. Others have confirmed the important role of this region in writing, although the observed dysfunction was not always restricted to a pure agraphia [29–34]. On the other hand, the description of various forms of agraphia following lesions at other locations in the brain questioned the uniqueness of ‘Exner’s area’ in writing [35,36]. Furthermore, the work of Exner has been criticized, because he studied only a limited number of patients of whom documentation was not very accurate, while agraphia was often accompanied by other symptoms [37].

With the advent of fMRI, more detailed descriptions of the putative ‘Exner’s area’ became available. Most of these studies indicated that the left mFG, or premotor cortex, is a crucial brain region for writing [38–41], although subjects did not actually perform a writing task in all studies. Moreover, also the posterior segment of the left superior frontal gyrus (sFG) has been claimed to represent the frontal writing center [42], possibly in conjunction with the left supramarginal gyrus [43]. Opposed to the concept of a center exclusively involved in writing, it has been argued that the frontal (premotor) writing region is an area in which the representation of graphemes is embedded, which may thus easily facilitate the generation of a motor program to use specific graphemes in writing [29,44]. The results of functional brain imaging have highlighted other areas implicated in writing tasks such as the cortex around the intraparietal sulcus (IPS), the left angular gyrus, the left posterior inferior temporal gyrus (iTG), the supplementary motor area (SMA), pre-SMA, the left supramarginal gyrus and mid-cingulate cortex, bilaterally [38–43,45,46]. The fact that a pure agraphia is very rare suggests that the ability to write is a complex task relaying on an elaborate neuronal network. Moreover, from a theoretical perspective, it has been regarded unlikely that a specific region in the brain adopts a relatively recent cultural development, favoring the idea that the human brain must rely on existing structures in order to perform this complex task [47].

To summarize our above motivated hypothesis concerning hemisphere-specific motor functions underlying writing in right-handed subjects, we (i) expected a relatively larger contribution of the left PMv in writing with the right hand due to putative interactions with adjacent Broca's area and (ii) a stronger involvement of the right PMd in left-hand writing based on a right-hemisphere dominance for spatial movements particularly executed by proximal muscles. To provide support for the writing-specific character of this dissociation, we included a 'higher-order control’ task that required subjects to draw simple geometrical figures. This drawing task further enabled us to explore to what extent writing can be seen as either a complex manual skill or a unique kind of manual language performance. To gain optimal insight in sensorimotor transformations specifically involved in dictated writing and drawing, a tapping task was added to control for basic motor-related activations. Our design thus provided the opportunity to compare task-related responses in widely distributed cerebral circuitry, including parietal and temporal cortical regions, and thus assess whether writing and drawing elaborate or partly elaborate the same basic neuronal network.

## Materials and Methods

### Subjects

Sixteen healthy adult right-handed volunteers (9 female), mean age 26.8 years (SD 9.8 years), participated in this study. All had Dutch as a native language. The Edinburgh Handedness Inventory [48] confirmed that all subjects were right-handed with scores that varied between 65 and 100 (mean 88.4, SD 12.1). None of the subjects had neurological or psychiatric disorders and there were no lesions of upper extremities. They all signed an informed consent according to a protocol approved by the Medical Ethics Committee of the University Medical Center Groningen. Study procedures were explained one week before scanning and practiced briefly in a dummy MRI immediately before the experiment until subjects understood the tasks.

### Experimental procedure

The paradigm was constituted by six stimulus-response conditions and one rest condition. Subjects had to respectively write a short sentence with either the left or right hand (conditions 1 and 2), draw geometrical figures with either the left or right hand (3,4) and tap with the pencil in either hand (5,6). The task of drawing geometrical figures implied an advanced manual skill in using the pencil without linguistic involvement (apart from the auditory instructions), thus enabling the identification of writing-related activations, while tapping controlled for simple motor activations. During scanning, which was performed in a dark environment, subjects were positioned with pillows under their flexed knee, which enabled them to give stable support to a metal-free writing-case placed on their lap. With a pencil in either hand, they could comfortably write on a paper (size 28 x 32 cm) fixed on this ‘desk’, without actually seeing the result of writing.

**Fig 1:**
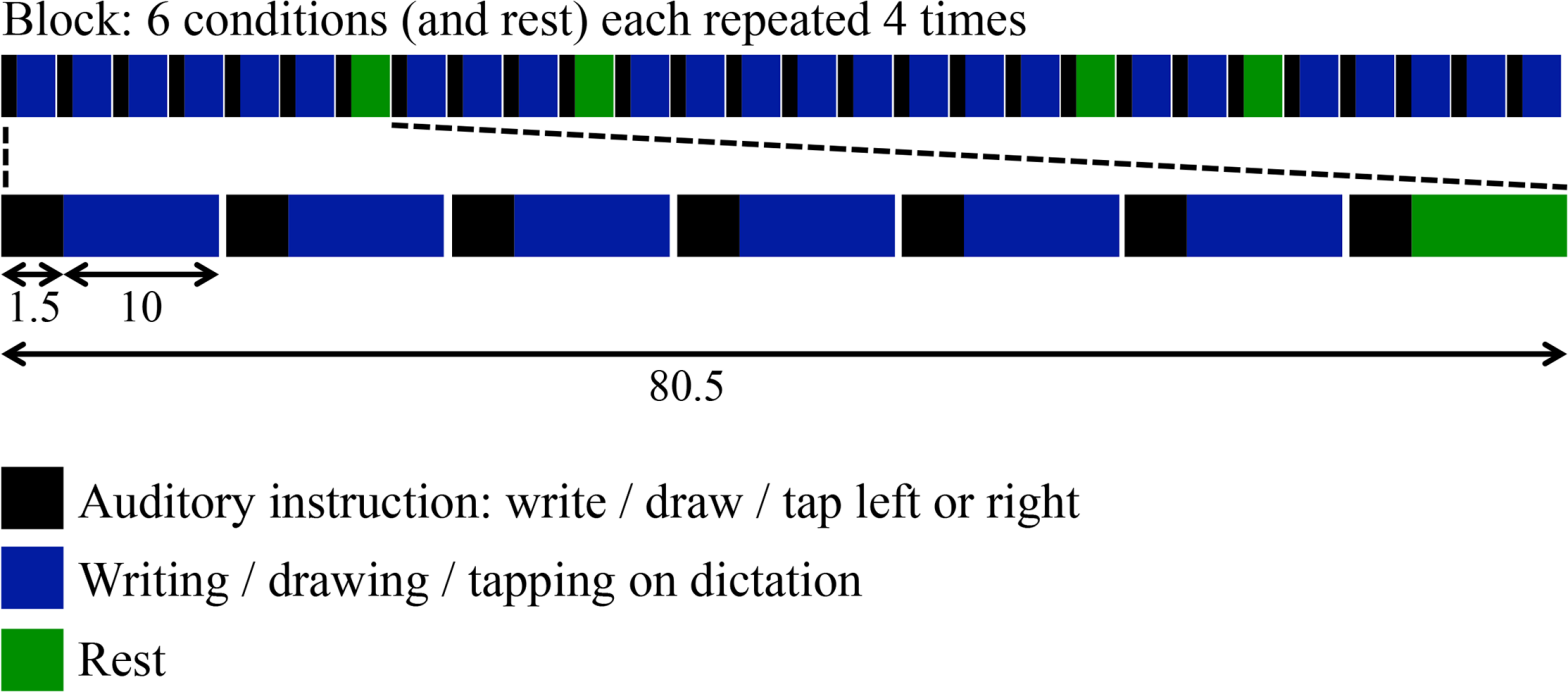
Scheme of the experimental paradigm, consisting of six different task conditions and one resting condition. The conditions were presented in a pseudorandomized order. The entire experiment consisted of eight different blocks, divided over two runs. Each condition was thus presented 32 times. There were no delays between different trials.

Subjects were instructed to write in cursive from left towards right and to draw figures in a similar order, as in normal writing. Conditions were aurally specified in 1.5 seconds via headphone by announcing e.g. ‘write left’ or ‘tap right’. For writing, subjects subsequently had to write easy sentences on dictation during 10 s (e.g. ‘the dog barks’ in Dutch). The aural instructions were slow enough for subjects to write subsequent words of a sentence on dictation. The drawing task was constituted by successive series of four randomly ordered geometrical figures (circle, oval, square, triangle) that were also aurally instructed. Tapping implied that subjects tapped with the pencil in response to a series of seven aural cues with random intervals. Subjects tapped when they heard ‘tap’ (in Dutch). In all three conditions the instructed performance allowed optimal filling of the ten second trials, without time pressure. The stimuli were presented in a block design, with eight different blocks equally divided over two runs (four trials of 11.5 s for the six conditions, see Fig 1). In each block every condition was presented four times. In this way all conditions were presented 32 times. The conditions were presented in a pseudo-randomized order using ‘Presentation’ (Neurobehavioural systems, Inc. Albany, USA). There were 32 different three-syllable sentences, 32 different combinations of figures and 32 tap trials with different intervals between cues. Half of the instructions concerned a first performance of the left hand and half concerned initial right-hand performance. The rest condition implied that subjects ‘hold pencils’ in the first 1.5 seconds and leave their hands on the writing-case without further action in the following ten seconds. During the whole experiment subjects held a pencil in both hands. Subjects were in the scanner for about 50 minutes. Between the two runs, a T1-weighted anatomical image was acquired and a new paper was placed on the writing-case. An infrared camera in the scanner room verified that the subjects actually performed the tasks.

### Data acquisition

Data acquisition was performed using a 3 T Philips MR system (Best, The Netherlands) with a 32-channel SENSE head coil. Functional images were acquired using a gradient-echo T2* Blood Oxygen Level Dependent (BOLD) technique using the following parameters: field of view 224 x 136.5 x 224 mm, TR = 2000 ms, TE = 28.0 ms, flip angle 70°, 39 slices without slice gap, isotropic voxels 3.5 x 3.5 x 3.5 mm, axial orientation, 650 volumes per run. A T1-weighted 3D anatomical scan was acquired to obtain high-resolution anatomical information with a field of view of 232 x 170 x 256 mm, TR = 9.0 ms, TE = 3.5 ms, flip angle 8°, 170 slices without slice gap, voxel size 0.9 x 1.0 x 1.0 mm.

### Data analysis

Image processing and voxel-based statistical analysis was conducted using Statistical Parametric Mapping [49], version 8 (2009, Wellcome Department of Cognitive Neurology, London, UK: http://www.fil.ion.ucl.ac.uk/spm). Preprocessing with SPM included realignment, coregistration with the anatomical image, normalization to the Echo Planar Image (EPI) of the Montreal Neurological Institute (MNI) brain and smoothing with a Gaussian filter of eight mm Full Width at Half Maximum (FWHM).

Cerebral activations were rendered on a standard MNI brain. All conditions were modeled in a block design at subject level for statistical analysis of regional differences in cerebral activations. We corrected for head motion, using regressors describing head motion that were included at subject level. These included three rotational and three linear movement parameters together with their quadratic, as well as the derivatives of these computations.

To identify the cerebral activations related to the different tasks, activity of the six conditions was contrasted with the rest condition. After that, the individual contrasts were compared at group level using one-sample t-tests. We assumed that the conditions were dependent with equal variance and subjects were assumed to be independent with equal variance. Writing and drawing conditions were contrasted with the tapping conditions to correct for simple motor activations. Clusters of increased activation were regarded statistically significant at p<0.05 (FWE corrected, cluster extent k = 8). In order to avoid false-negative results, an additional assessment was made at voxel-level threshold p<0.001 (uncorrected, k = 8) to identify possible clusters that might additionally reach statistical significance corrected for the entire brain volume. Given our hypothesis concerning writing-related activations in the PMv and PMd of the two hemispheres [19], it was also used to identify activations in these regions in case no effect was seen at the FWE-corrected level. To specifically test whether the presence of a differential contribution of either the PMv or PMd to writing, we performed a region of interest (ROI) analysis on these areas in each of the two hemispheres. ROI’s were derived from an existing human motor area template [50]. We extracted the mean regional beta values using these ROI’s. To compare the differential contribution of the PMv and PMd we calculated a ratio per subject using the following formula: Beta values PMd / (beta values PMd + beta values PMv). For writing with the right hand we calculated this ratio in the left hemisphere and for writing with the left hand in the right hemisphere. This allowed us to test the differential contribution of the PMv and PMd to writing. We compared the mean ratios of right- and left-hand writing with a paired samples t-test with IBM SPSS Statistics version 20. This was also done for the drawing task. Differences were considered statistically significant when p<0.025 (Bonferroni correction).

## Results

**Fig 2:**
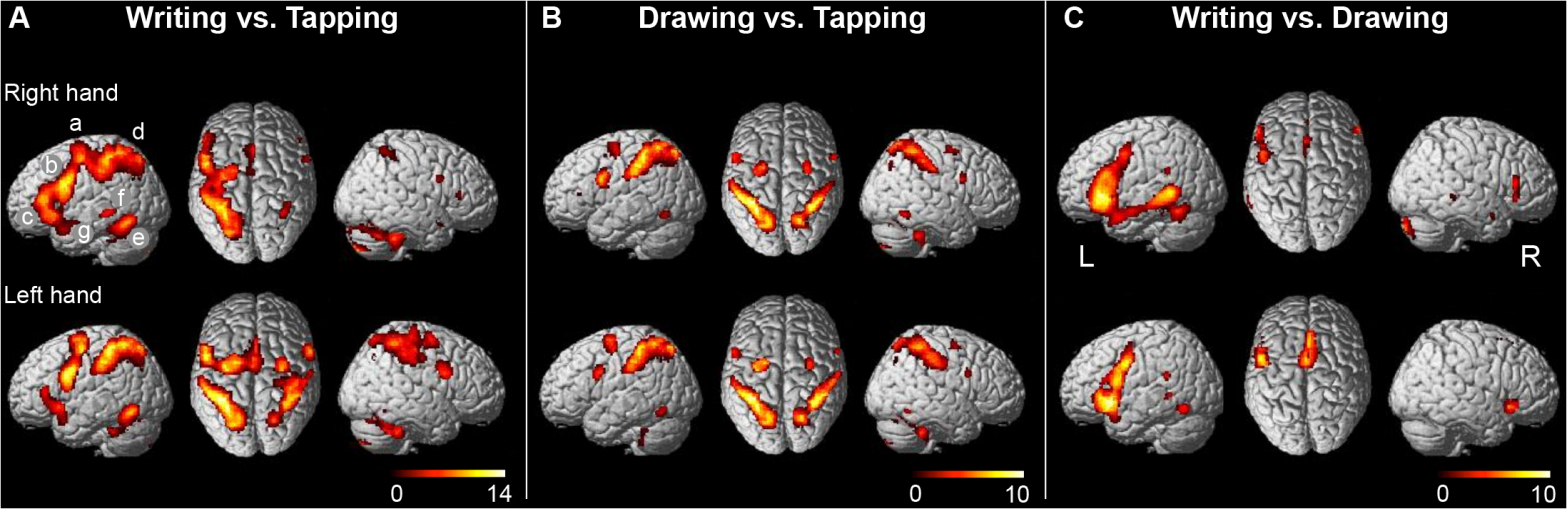
(A) Cerebral activations for right- and left-hand writing versus respectively right- and left-hand tapping. (B) Cerebral activations for right- and left-hand drawing versus respectively right- and left-hand tapping. (C) Cerebral activations for right- and left-hand writing versus right- and left-hand drawing respectively. The presented activations result from analyses using a statistical threshold of p<0.05 FWE corrected, with an extended voxel threshold (k) of 8 voxels. Clusters are rendered onto the surface of a standard anatomical brain volume (Montreal Neurological Institute, MNI). Coordinates and T-values are reported in Tables 1 and 2. L = left hemisphere of the brain, R = right hemisphere of the brain, a = dorsal premotor cortex, b = ventral premotor cortex, c = Broca’s area, d = parietal cortex, e = posterior part of the inferior temporal gyrus, f = posterior part of the middle temporal gyrus, g = anterior superior temporal sulcus.

Contrasting the higher-order motor conditions writing and drawing to simple tapping revealed characteristic distributions of activation that predominantly included parietal and premotor cortical regions. At first sight, a strong left-hemisphere dominance was seen in writing, not only when performed with the right but also with the left hand (Fig 2A) while for drawing (contrasted to tapping) a rather symmetrical parietal-premotor pattern was revealed, regardless of the hand of action (Fig 2B). These condition-related activations included local maxima in the PMd and PMv, at coordinate positions fitting the premotor templates of Mayka et al. [50]. To answer our first question concerning hemisphere-specific differences in PMd and PMv contributions to contralateral writing, differences between the writing-related activations were analyzed.

### Differential contribution of the PMv and PMd to left- and right-hand writing

**Fig 3:**
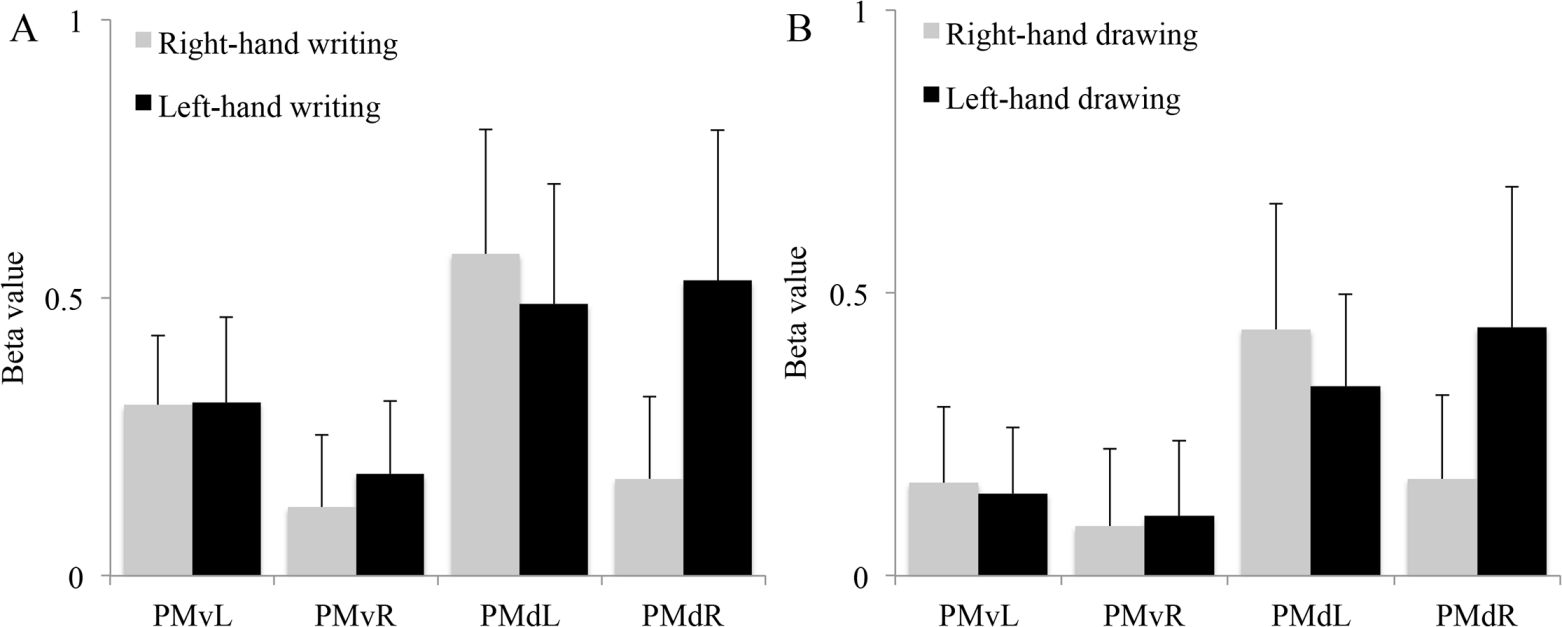
Activations of the left and right PMv and PMd during writing (A) and drawing (B) with the right and left hand. Beta values represent the mean activation over all subjects. The error bars are the standard deviations. PMvL = left ventral premotor cortex. PMvR = right ventral premotor cortex. PMdL = left dorsal premotor cortex. PMdR = right dorsal premotor cortex.

Analysis of the four pre-defined ROI’s revealed that for respectively the left PMv, right PMv and left PMd activations related to either right- or left-hand writing were highly similar, while right PMd activation was stronger during left- than right-hand writing (Fig 3A). Moreover, activation was stronger in the left than in the right PMv for writing with either hand. This hemisphere difference in relative contributions of the PMv and PMd to writing was statistically substantiated by a significant difference between the mean activation ratios [PMd / (PMd+PMv)] of 0.65 (SD 0.10) for the left hemisphere in right-hand writing and 0.78 (SD 0.20) for the right hemisphere in left-hand writing (p = 0.021) (Fig 3A). The ratios for the two hemispheres did not differ for the figure drawing task, with mean ratios of 0.82 (SD 0.35) for the left hemisphere in right-hand drawing and 0.75 (SD 1.3) for the right hemisphere in left-hand drawing (p = 0.77) (Fig 3B). The absence of significance in the latter descriptively suggests that the relatively strong right PMd contribution to left-hand writing was writing-specific although we acknowledge that we did not provide statistical support for such specificity. The plots in Fig 3 are consistent with the patterns of activation in Fig 2C, showing increased activation of the left PMv during both left- and right-hand writing when directly compared to drawing.

### Writing contrasted to tapping

**Fig 4:**
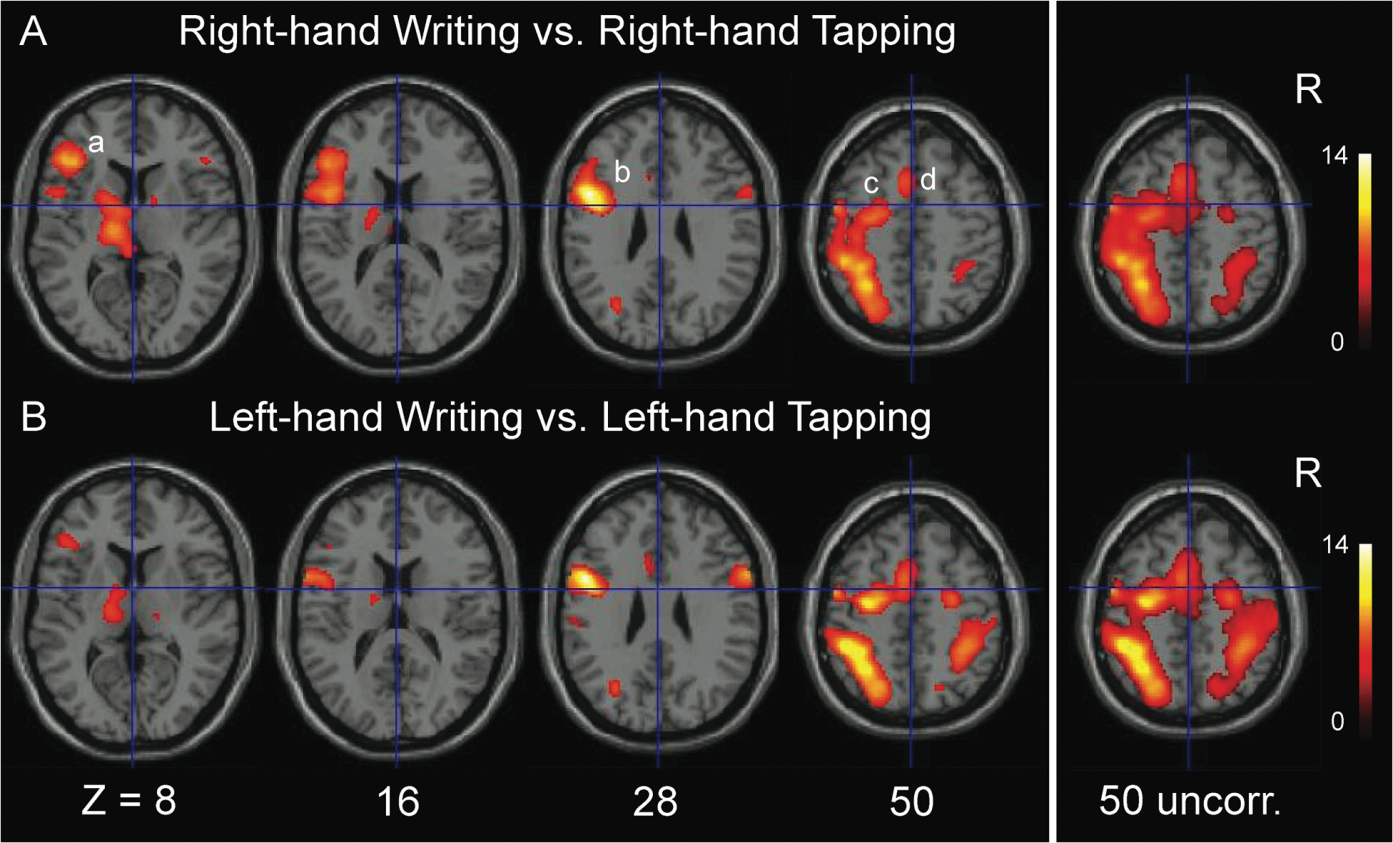
Cerebral activations for right- and left-hand writing versus respectively right- and left-hand tapping (p<0.05 FWE corrected, with an extended voxel threshold (k) of 8 voxels). Clusters are projected on transversal sections of a standard anatomical brain (MNI). The z coordinate indicates the distance to the plane traversing the anterior-posterior commissures in mm. The uncorrected coordinate is at p<0.001 with an extended voxel threshold (k) of 8 voxels. R = right side of the brain (neurological convention). a = Broca’s area, b = ventral premotor cortex, c = dorsal premotor cortex, d = pre-supplementary motor area.

In the next section we describe the details of the distributed activations related to writing with either the right or left hand. Contrasting right-hand writing with tapping of the same hand resulted in significant activations (p<0.05, FWE corr.) in the left hemisphere with an extensive confluent cluster, within which local maxima could be discerned identifying the PMd, PMv, and putative Broca’s area in the inferior frontal gyrus (iFG) (Figs 2A and 4A). This left-hemisphere cluster further extended posteriorly along the IPS. In the right hemisphere, significant activations were less robust with separate clusters in the PMv, the mirror equivalent of Broca and along the horizontal segment of the IPS, respectively. Right PMd activation was not significant at this FWE-corrected threshold, although it was identified at voxel-level p<0.001 (uncorr.) (Fig 4A). In addition, two foci of left temporal cortex activation were seen, located on the posterior part of the middle temporal gyrus (mTG) and iTG, respectively. The posterior part of the mTG activation extended over its dorsal surface into the superior temporal sulcus on its superior surface. Activations restricted to only the left hemisphere were also found in the supplementary motor area, pre-SMA, cingulate cortex and thalamus. In the basal ganglia and cerebellum, activations were bilaterally distributed. Coordinates of significant activations are further specified in Table 1.

**Table 1:** Cerebral activations related to writing compared to tapping.

Brain region (BA)	Left				Right			
	x	y	z	T-value	x	y	z	T-value
*Right-hand writing versus right-handed tapping*								
iFG (44/45)	-44	30	12	9.4	50	30	10	5.6
Anterior sTG (38)	-52	12	-22	8.6				
PMv (6)	-48	8	28	14.3	60	10	28	6.0
PMd (6)	-24	-8	50	7.6				
Superior parietal lobe (7)	-30	-52	68	7.5	34	-52	60	5.5
Inferior parietal lobe (40)	-30	-54	50	10.1	34	-46	52	5.3
iTG (20)	-54	-62	-16	10.0				
mTG (21)	-54	-36	-4	6.9				
Pre-SMA (6)	-6	16	48	6.9				
SMA (6)	-4	-4	56	5.8				
Cingulate cortex (32)	-6	20	30	5.1				
Basal ganglia / Anterior thalamus	-14	0	8	7.1	14	4	4	5.5
Posterior thalamus	-14	-16	6	8.5				
Cerebellum	-18	-66	-28	6.4	14	-52	-22	9.6
					16	-74	-50	12.2
*Left-hand writing versus left-hand tapping*								
iFG (44/45)	-42	30	4	6.4				
Anterior sTG (38)	-52	12	-20	7.1				
PMv (6)	-50	6	30	14.6	60	10	28	9.1
PMd (6)	-30	-10	52	11.6	30	-8	54	7.9
Superior parietal lobe (7)	-20	-70	56	9.7	18	-72	62	7.1
Inferior parietal lobe (40)	-36	-44	52	11.9	38	-48	56	8.3
iTG (20)	-52	-62	-14	11.3	52	-56	-16	5.1
Pre-SMA (6)	-8	0	68	5.0				
SMA (6)	-4	-2	54	9.3				
Cingulate cortex (32)	-6	16	42	7.1				
Basal ganglia / Anterior thalamus	-14	6	2	5.2				
Posterior thalamus	-14	-16	6	7.0	16	-16	4	5.3
Cerebellum	-18	-68	-24	7.7	26	-64	-26	13.0
	-16	-68	-50	6.2	16	-74	-50	12.0

The coordinates and T-values of local maxima within significant clusters are reported (p<0.05 FWE corrected, with an extended voxel threshold (k) of 8 voxels). Positive x, y and z coordinates indicate respectively coordinates right, anterior and superior of the middle of the anterior commissure. BA = Brodmann area. MNI = Montreal Neurological Institute. sTG = superior temporal gyrus. iFG = inferior frontal gyrus. iTG = inferior temporal gyrus. mTG = middle temporal gyrus. SMA = supplementary motor area.

The pattern of significant activations related to left-hand writing, contrasted with left-hand tapping (p<0.05, FWE corr.), showed strong resemblance to the right-hand writing pattern (Figs 2A, 4A and 4B). In left-hand writing, left-hemisphere activations were also stronger than activations in the right hemisphere. Now, the left premotor cluster of PMv and PMd activations was separated from Broca’s area and the parietal foci of activations. In contrast to right-hand writing, left-hand writing was related with significant right PMd activation together with activation of the right PMv, while the spatial extension of right parietal activation was larger in left- than in right-hand writing. No activation was seen in either the left or right mTG or right iFG. See Table 1 for a further summary of activations. At relaxed voxel-threshold of p<0.001 (uncorr., k = 8), no additional clusters were found that reached statistical significance when corrected for the entire brain volume.

### Drawing contrasted to tapping

The patterns of activation that resulted from the comparison of drawing with tapping showed a clear overlap with the writing-related activations, although characteristic differences were also observed. The PMv and PMd were bilaterally activated during both right- and left-hand drawing, while activations around the IPS were more symmetrically than in writing (Fig 2B). Irrespective of the hand used for drawing, activations in the left hemisphere were stronger than in the right hemisphere. iTG activation was seen bilaterally, without activation of the mTG. Neither iFG nor pre-SMA activation was seen. The only significant activation in the basal ganglia was in the left anterior putamen during right-hand drawing (contrasted to right-hand tapping). At p<0.001 (voxel-level uncorr., k = 8), this anterior putamen activation extended medially in the left pallidum and thalamus. The locations of the drawing-related activations are listed in Table 2. Comparing right-hand drawing with right-hand tapping at an initial voxel-threshold of p<0.001 (uncorr., k = 8) revealed one additional cluster that reached statistical significance (brain-volume corrected), which was located in the left iFG at [x -38, y 32, z 10] (p = 0.013).

**Table 2:** Cerebral activations related to drawing compared to tapping.

Brain region (BA)	Left				Right			
	x	y	z	T-value	x	y	z	T-value
*Right-hand drawing versus right-handed tapping*								
PMv (6)	-50	8	28	9.6	60	10	28	6.7
PMd (6)	-28	-8	52	7.8	28	-4	54	6.4
Superior parietal lobe (7)	-16	-70	56	8.6	16	-72	62	7.5
Inferior parietal lobe (40)	-46	-38	44	10.5	38	-38	46	8.8
iTG (20)	-52	-62	-14	6.2	52	-56	-14	6.1
Striatum	-22	10	6	5.3				
Cerebellum	-18	-68	-26	5.8	24	-52	-30	7.5
	-18	-70	-50	5.3	16	-74	-50	10.8
*Left-hand drawing versus left-hand tapping*								
PMv (6)	-50	8	28	7.6	60	10	28	6.2
PMd (6)	-30	-10	52	8.4	28	-6	52	6.3
Superior parietal lobe (7)	-18	-70	56	8.8	16	-72	62	7.2
Inferior parietal lobe (40)	-36	-44	50	9.7	38	-38	48	8.3
iTG (20)	-52	-62	-14	6.4	54	-58	-14	5.5
Cerebellum	-20	-68	-26	6.5	26	-64	-26	9.4
	-16	-66	-50	6.1	14	-74	-50	9.6

The coordinates and T-values of local maxima within significant clusters are reported (p<0.05 FWE corrected, with an extended voxel threshold (k) of 8 voxels). Positive x, y and z coordinates indicate respectively coordinates right, anterior and superior of the middle of the anterior commissure. BA = Brodmann area. MNI = Montreal Neurological Institute. iTG = inferior temporal gyrus.

### Writing contrasted to drawing

**Fig 5:**
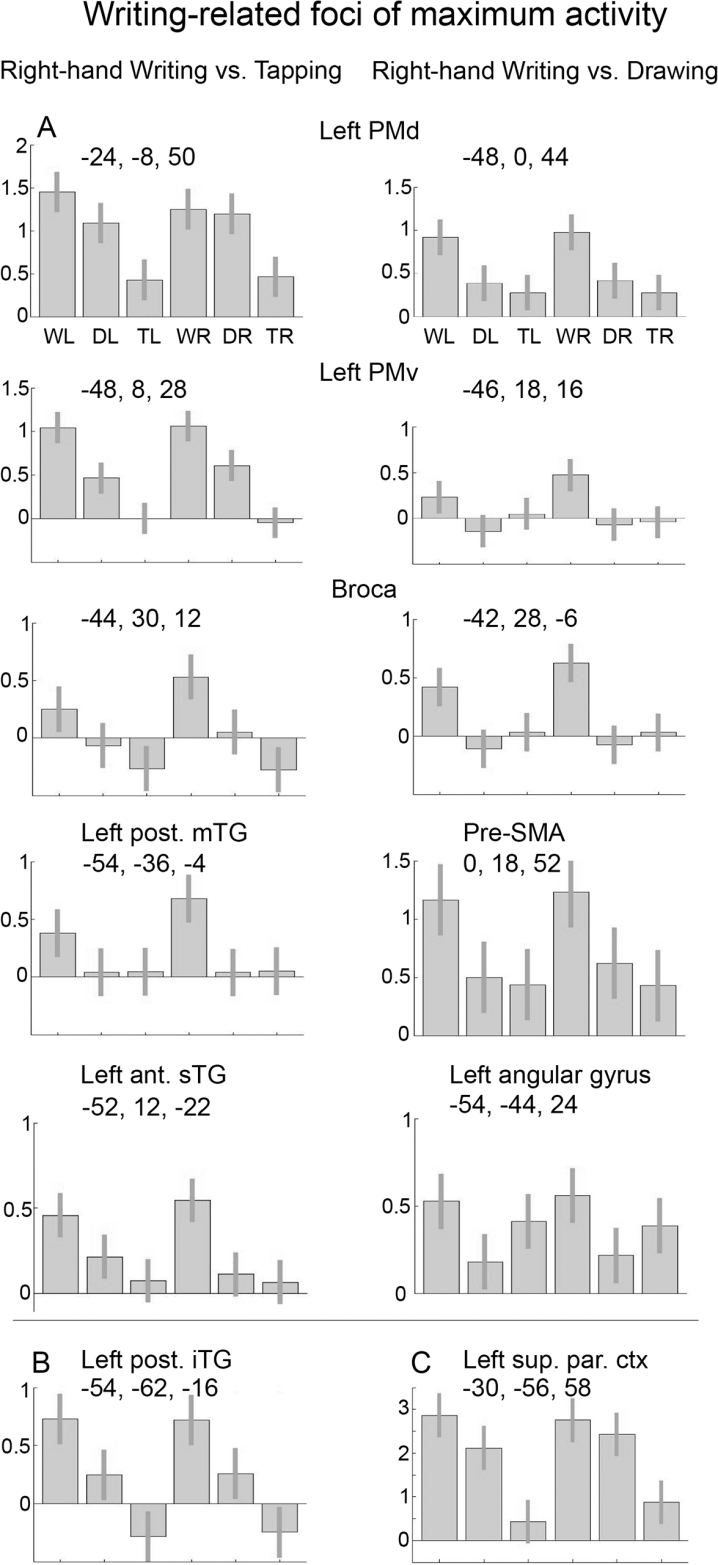
Writing-related foci of maximum activity. (A) Contrast estimates with 95% confidence intervals from writing-related foci of maximum activation from the contrasts right-hand writing versus right-hand tapping and right-hand writing versus right-hand drawing, as reported in Tables 1 and 3. (B) Contrast estimates with 95% confidence intervals from the focus of maximum activation in the left inferior temporal gyrus from the contrast of right-hand writing versus right-hand tapping (for the same condition with the left hand this focus was at -52, -62, -14). (C) Contrast estimates with 95% confidence intervals from the focus of maximum activation of the left parietal cortex (coordinates from Brownsett and Wise, 2010). WL = left-hand writing, DL = left-hand drawing, TL = left-hand tapping, WR = right-hand writing, DR = right-hand drawing, TR = right-hand tapping, iTG = inferior temporal gyrus, mTG = middle temporal gyrus, sTG = superior temporal gyrus.

To gain more insight in the actual writing-related character of the identified regions, the writing-related activations were contrasted to drawing. For both the right and left hand this resulted in activations strongly lateralized to the left hemisphere (Fig 2C). The resulting foci of maximum activation in the PMd and PMv were each located at a slightly more antero-inferior location relative to the maximum activation identified by right-hand writing contrasted to right tapping (Fig 2A and 2C, coordinates in Tables 1 and 3 and Fig 5A). Although the writing-related effects were stronger at the local maxima obtained by the latter contrast, a considerable drawing-related effect was also seen at these foci (Fig 5A). For the left mTG activation, the focus of maximum activation related to right-hand writing (versus tapping) was at the same location as the maximum that resulted from the comparison with drawing, underscoring its strong writing-related involvement without a contribution to drawing (Fig 5A). The left iTG was active in both writing and drawing, but more pronounced during the writing task. There was a stronger activation in the anterior part of the left superior temporal gyrus (sTG) during writing with either hand compared to drawing and tapping (Figs 2A, 2C and 5A). Also, activation in the left angular gyrus was more profound during writing compared to drawing, but not compared to tapping (Figs 2A, 2C and 5A). See Table 3 for a summary of activations.

**Table 3:** Cerebral activations related to writing compared to drawing.

Brain region (BA)	Left				Right			
	x	y	z	T-value	x	y	z	T-value
*Right-hand writing versus right-handed drawing*								
iFG (44/45)	-42	28	-6	11.6	42	28	-6	5.5
Anterior STG (38)	-52	10	-22	9.4	58	4	-24	5.6
PMv (6)	-46	18	16	8.3				
PMd (6)	-48	0	44	7.3				
Angular gyrus (39)	-54	-44	24	5.9				
iTG (20)	-50	-58	-22	6.4				
mTG (21)	-60	-46	0	10.0	48	-38	-2	6.2
Pre-SMA (6)					0	18	52	5.4
Basal ganglia / Anterior thalamus	-8	-2	4	6.0				
Thalamus	-10	-16	8	5.5				
Cerebellum					16	-86	-32	7.0
					14	-50	-20	5.5
*Left-hand writing versus left-hand drawing*								
iFG (44/45)*	-40	28	-4	9.4	34	22	-6	6.6
PMv (6)	-48	4	30	9.6				
PMd (6)	-52	-2	44	8.3				
Angular gyrus (39)	-52	-44	22	6.6				
iTG (20)	-50	-62	-14	6.4				
mTG (21)	-58	-46	2	5.4				
Pre-SMA (6)					0	4	60	7.9
Basal ganglia / Anterior thalamus	-8	0	4	6.2	8	0	6	5.3
Thalamus	-14	-4	-10	5.7				
Cerebellum					28	-64	-26	5.4

The coordinates and T-values of local maxima within significant clusters are reported (p<0.05 FWE corrected, with an extended voxel threshold (k) of 8 voxels). Positive x, y and z coordinates indicate respectively coordinates right, anterior and superior of the middle of the anterior commissure. BA = Brodmann area. MNI = Montreal Neurological Institute. sTG = superior temporal gyrus. iFG = inferior frontal gyrus. iTG = inferior temporal gyrus. mTG = middle temporal gyrus. SMA = supplementary motor area.

*The left iFG cluster was confluent with activation in the left anterior STG.

Although significant pre-SMA activation only occurred during writing and not drawing, each contrasted to tapping (Fig 2A and 2B and Tables 1 and 2), it was its anterior segment that was most specifically involved in writing, independent whether this concerned right- or left-hand writing (Fig 5A). Finally, response profiles in the left iTG showed that this region was more activated in writing than in drawing but that the activation was not writing-specific (Fig 5B). Given the previously described role of the left superior parietal cortex in writing [51], we assessed the effects at the location they reported and indeed found a strong writing-related effect (compared to tapping) which was, however, not significantly larger than that in drawing (Fig 5C). At relaxed threshold (p<0.001, uncorrected), this contrast revealed an increase of activation in the left superior parietal cortex [x -30, y -72, z 44] which did, however, not reach cluster-level significance corrected for the entire brain volume (p = 0.29).

### Cerebellar and subcortical activations

**Fig 6:**
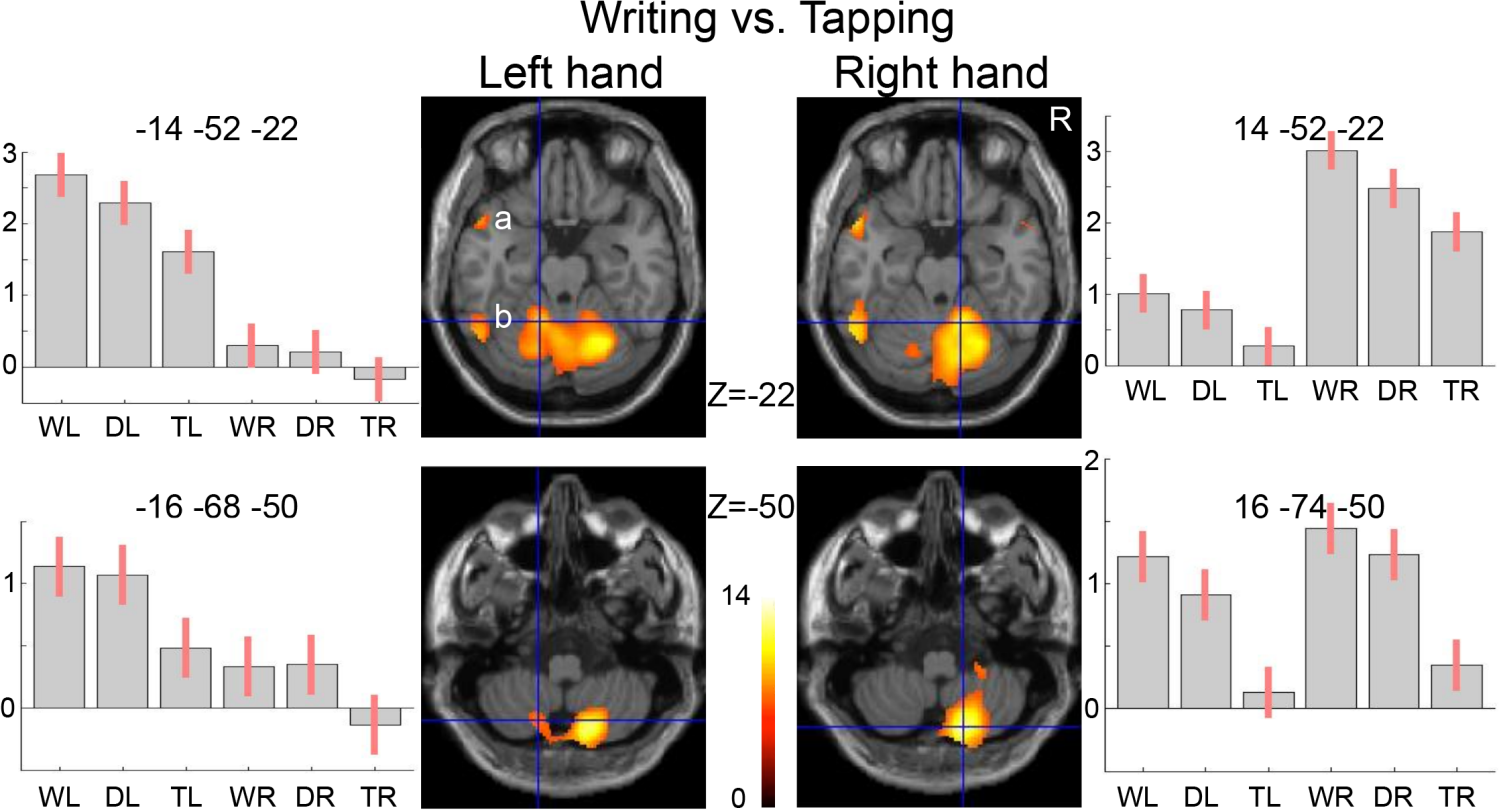
Cerebral activations for right- and left-hand writing versus respectively right- and left-hand tapping (p<0.05 FWE corrected, with an extended voxel threshold (k) of 8 voxels) for the cerebellum clusters in the anterior lobe (upper row) and posterior lobe (lower row). Plots demonstrate the activation during the six different tasks with the 95% confidence interval. Clusters are projected on transversal sections of a standard anatomical brain (MNI). The z coordinate indicates the distance to the plane traversing the anterior-posterior commissures in mm. R = right side of the brain (neurological convention). a = anterior superior temporal sulcus, b = posterior part of the inferior temporal gyrus.

In the cerebellum, the location of activations pointed at a characteristic functional difference between its anterior and posterior lobes. Activations in the anterior lobe were ipsilateral to hand movement, irrespectively whether it concerned writing, drawing or tapping while posterior lobe activation was particularly related to the higher-order motor tasks writing and drawing. Moreover, posterior cerebellum activation was most pronounced in the right lobe, opposite to the dominant left cerebral hemisphere activations, irrespective of writing or drawing with either the right or left hand (Fig 6). In the basal ganglia, the confluent cluster of left thalamus and pallidum activation during right-hand writing (contrasted to right-hand tapping) extended in the anterior segment of the left putamen (Fig 4), whereas left-hand writing (compared with left-hand tapping) was neither related with right nor with left anterior putamen activation. This absence remained at relaxed threshold (p<0.001 voxel-level uncorr.). Compared to rest, all three motor tasks were related with contralateral activation of the posterior putamen.

## Discussion

In this study we aimed to identify cerebral activations related to writing, balanced for motor functions also implicated in drawing and tapping with a pencil, while hemisphere-specific contributions were assessed by writing with each hand. In this way, we were able to distinguish two levels of writing-related activations concerning the identified brain regions. We found (i) regions that were uniquely involved in writing and (ii) regions that were involved in both writing and drawing, contrasted to tapping, but with a significantly stronger contribution to writing than to drawing. Five left-hemisphere areas were implicated exclusively in writing, being the antero-inferior parts of both PMv and PMd, Broca’s area, the posterior part of the left mTG and the pre-SMA. Activation of the superior parietal cortex was not unique for writing because this area was also strongly involved in the drawing task. We further obtained support for our specific 'movement' hypothesis concerning a differential involvement of the PMv and PMd in the two hemispheres.

It appeared that, while the PMd of each hemisphere contributed equally to contralateral writing, the left PMv made a stronger contribution to right-hand writing than the right PMv to writing with the left hand. As a consequence, the right PMd was relatively stronger involved in left-hand writing. We did not gain formal statistical support that this right PMd characteristic was writing-specific, compared to drawing. The dominant role of the left PMv in writing is consistent with the view that it has an intimate relation with Broca’s area and subserves motor integration with frontal language circuitry, irrespective which hand is used. As a common characteristic in writing and drawing, we found bilateral overlap between parietal-premotor activations in the two conditions, regardless the hand of execution. On the other hand, a characteristic difference between these two tasks was limb-independent dominance of such activations in the left hemisphere during writing, while for drawing a more symmetrical pattern was seen. This symmetrical pattern in drawing, contrasted to tapping with a pencil, indicates that the conditions in our study were sufficiently balanced for generic sensory and motor functions. The bilateral absence of primary sensorimotor activation in writing contrasted to drawing with each hand, as well as the absence of any right premotor activation in the same contrast for left-hand performance further support this conclusion.

### Segregated processing streams

The enhanced recruitment of parietal-premotor networks during writing, compared to tapping, points at general features of higher-order motor control [52]. In this, parietal-premotor circuitry computes sensorimotor transformations for goal-directed movement along pathways that are, to some extend, functionally segregated. E.g., a dorsal-ventral segregation between two processing streams can be discerned in which interconnection between the PMd and postero-superior parietal cortex particularly subserves target-directed spatial navigation [5,14,53–55], while a network comprising the PMv and antero-inferior parietal cortex is particularly involved in the integration of object shape, (predicted) touch and prehension [13,56,57]. Such dorsal-ventral distinction runs parallel with the contributions of the PMd and PMv to proximal and distal upper limb movements, respectively, not only reflected by their spatial relationships with the somatotopic representation of the primary sensorimotor cortex, but also by their direct output to the spinal cord. In this, the PMd projects to spinal cord segments corresponding to mostly proximal arm movements while the PMv connections are restricted to segments that particularly control distal hand movements [3]. The dominance of the right PMd, relative to the PMv, we found in left-hand writing is thus consistent with the more proximal arm movements made during writing with the left hand, compared to writing with the right hand [18,19].

Considering the consistent role of premotor regions in (partially) segregated circuitries supporting sensorimotor transformations, we propose that three of such processing streams may be inferred from the distribution of writing-related activations in our data. Parallel to the pathways listed above, functional circuitry comprising the anterior PMv and exclusively the left posterior mTG might logically support audition-based language-to-motor transformations. The overall motor act of writing would thus result from convergence of three main pathways originating from posterior cortical regions.

This implies that in writing, the dorsal network comprising the PMd and superior parietal cortex provides a spatial reference frame enabling the horizontal alignment of successive letters and words in a sentence. This is consistent with the previously described posterior parietal activation in writing, explained as a kinematic representation of graphomotor trajectories [58]. Spatial ordering is also required for drawing, fitting the parietal activations we found during this task. Writing may even be seen as a kind of figure drawing, except for the fact that a specific meaning is coded by the arrangement of letter figures. The efficiency of a horizontal letter order for attributing such meaning to words (i.e. adding semantic value) may point at an enhanced recruitment of this spatial function in writing, indeed reflected by stronger writing-related activation in particularly the left posterior parietal cortex, relative to drawing.

In the model of three parallel processing streams, the second network of coherent interconnection between the PMv and antero-inferior parietal cortex logically underlies effective use of the pencil in a body-centered coordinate system [54,59,60]. Such complex tool use holds for both writing and drawing. Overlap in the related patterns of cerebral activations supports the evolutionary viewpoint that it seems efficient to relay on existing instead of entirely different networks for writing [47]. On the other hand, the pronounced left-hemisphere dominance of this ventral parietal-premotor circuitry in writing, more than in drawing, may illustrate that writing requires an additional level of complexity in neuronal processing. This is consistent with case reports of agraphia without disturbances in the use of tools [61].

Equivalent to neuronal mechanisms underlying sensorimotor transformations in the other two processing streams, the putative pathway linking the anterior segment of the left PMv with the posterior part of the left mTG was particularly related to writing, not involved in drawing. This pathway seems optimally placed to funnel auditory language information to circuitry that organizes the motor action of writing guided by dictation via the extreme capsule and/or arcuate fasciculus [62,63]. These perisylvian activations did not fully coincide with the general language system interconnecting the left superior temporal gyrus (Wernicke’s area) and Broca’s area in the iFG of the same hemisphere [64]. Another argument against general language involvement is the response profile with stronger activations during right- than during left-hand writing. This supports the view that these left-hemisphere activations represent the efficiency of right-hand writing and not covert speech, as the latter would be expected to similarly accompany right and left-hand writing (see further the paragraph below treating the temporal cortex).

The previous paragraphs support the concept that writing is a cerebral function that arises according a general organization principle of neuronal network processing implicated in sensorimotor transformations, in which both segregation and integration of information streams can be discerned. On the other hand, particular brain regions appear to play a highly dominant role within such networks. The apparent occurrence of pure agraphia due to a focal left premotor cortex lesion (‘Exner’s area’) provides support for such crucial network nodes. In the next paragraphs, writing-related activations will therefore be discussed with emphasis on the specific contributions of premotor, parietal and temporal cortex regions, respectively.

### Premotor cortex in writing

Both the PMd and the PMv of the left hemisphere contributed to writing with each hand. At the focus of maximum PMd activation, the magnitude of responses for writing and drawing were virtually the same while at the focus of maximum PMv activation, the effect of writing was about twice as strong as that of drawing. This suggests a more specific involvement of the PMv in writing. On the other hand, when the pattern of activations during writing was contrasted to drawing, exclusively writing-related activations within both the PMd and PMv were identified at more antero-inferior locations. While the robust activations at this second PMd focus remained similar for right- and left-hand writing, the left anterior PMv response was larger for right- than for left-hand writing with a profile that resembled that of a third cluster at Broca’s area. The strong association of these two regions with particularly right-hand writing and to lesser extend left-hand writing, may reflect the efficient integration of manual skill and language in writing. Such common function is consistent with the cytoarchitectural similarity of these adjacent cortical regions [16,17].

Writing-specificity of the antero-inferior part of the left PMd held for both hands. This may be an argument for its contribution to general orthographical (grapheme) construction in writing, with indeed the consequence that a lesion at this location results in a failure of such graphemic motor function of each hand. The antero-inferior PMd location at the junction of the mFG and sFG is consistent with the functional imaging locations reported for Exner’s area (summarized in Planton et al. [45]). While Planton et al. calculated mean PMd coordinates [x -22, y -8, z 54] for this putative Exner's area, variation within the PMd demarcation was acknowledged [45,50]. Such variation can often be attributed to differences in experimental design. E.g., letter drawing compared to imagining of letters activated the posterior part of the left PMd [65]. PMd activation in the study of Katanoda et al. extended e.g. between z-coordinates 58 and 64, using a visually-cued writing task, controlled for naming and tapping [43], while Roux et al. reported a more inferior PMd activation [x 26, y 0, z 43] during word dictation with control tasks of drawing circles and repeating a single syllable, respectively [66]. Consistent with our results, it seems that when adequately controlled for drawing, a more writing-specific function can be identified in the antero-inferior segment of the left PMd. Such functional parcellation within the PMd, as well as in the PMv, is consistent with previous findings of Schubotz and co-workers [67].

### (pre-)SMA in writing

We found significant writing-related activation of particularly the left SMA and pre-SMA, contrasted to tapping, while the responses related to drawing equaled that of tapping. This involvement in writing is consistent with previous functional imaging studies [45]. The SMA and pre-SMA are involved in preparing complex movement sequences [68], which may be an argument to consider the activation of these areas during our writing tasks as merely motor-related [45]. On the other hand, the (pre-)SMA has been proposed to play a similar role in motor and cognitive processing [69]. Along that line of reasoning, one may infer that this activation represents the increased level of sequential ordering of letters and words in writing a sentence, a mechanism not implicated in drawing. The (pre-)SMA would thus contribute to an equivalent neuronal mechanism in language and motor control. We think it is less plausible that the (pre-)SMA activation represents a non-specific enhancement of cognitive demand in writing, compared to drawing. Although the medial (pre-)frontal cortex indeed has a prominent role in action monitoring and decision making [70,71], writing in our study was instructed by dictation, without the necessity of making (free) choices. Moreover, activations during drawing and tapping were virtually the same in the (pre-)SMA, providing another argument against the explanation that the (pre-)SMA involvement in writing reflected increased general cognitive demand in writing.

### Parietal cortex in writing

Strong activation of particularly the left parietal cortex in writing of each hand points at a stronger left than right parietal contribution to writing in our study. Such limb-independent left parietal lateralization is consistent with the fMRI results of Sugihara and co-workers who asked subjects to write letters in the air with the index finger of each hand, while silent naming was used as a control condition [42]. The left parietal activation during writing in our study was, however, not significantly stronger than in drawing. Particularly for the superior parietal cortex, this is an important observation complementing the interpretation of previously reported superior parietal contributions to writing. The studies of both Menon and Desmond and Brownsett and Wise on this topic did not include drawing as a control task either [39,51]. Our results thus underscore that these previous results were not necessarily writing-specific. On the other hand, Beeson et al. did identify increased superior parietal activation relative to drawing [38]. However, in contrast to our drawing task, they employed a drawing task of repeatedly making similar circles, not specified by a distinct instruction. The observed increase in left superior parietal activation by Segal and Petrides during writing when controlled for reading and word retrieval as well as circular loop movements [72], evidently indicated parietal involvement in the higher-order motor aspects of the task, while functional interconnectivity characteristics supported interaction with cortical language regions. Similar to the Beeson study [38], loop movements controlled for the motor component of writing in the study of Segal and Petrides. The left-dominant parietal activation during writing with either hand in our study is consistent with such writing-related function. However, the absence of a writing-related increase of parietal activation when contrasted to drawing a series of various elementary figures, specified by auditory instructions, underscores that this region serves a more general audition-motor transformation, indeed concerning movements beyond the simple execution of a stereotypic movement pattern. This aspect of audition-guided drawing did apparently recruit parietal processing at a similar level as writing by dictation.

These findings fit the basic superior parietal role to provide an interface for the conversion of visual and auditory sensory information into body- and world-centered spatial coordinate frames [53]. The profile of activations in various parietal regions described by Brownsett and Wise further supports this view with superior parietal responses that were related exclusively to writing and not to speech or number assessment [51]. The spatial characteristics of writing, i.e. using orthographical information for arranging a well-ordered written text might thus be achieved in parallel with a segregated processing stream adding a meaningful content to such text. A consequence of spatial disorientation due to a discrete left superior parietal damage may indeed be optic ataxia associated with agraphia [39,73].

It was intriguing to see that activation at the left temporal-parietal junction, i.e. the left angular gyrus, was significantly increased in both writing and tapping compared to drawing, independent from the hand of action. An explanation for this common involvement in writing and tapping, and not in drawing, remains rather speculative. One might, in this respect, consider a relation with the basic role of the angular gyrus in early-stage motor intention, driven by either external or intern 'signals' [74–76]. In our study, particularly the immediacy of responses matching the aural information in both dictation and the verbal cues to 'tap' may suggest a common mechanism in the writing and tapping conditions facilitating efficient preparation of serial order, while the instruction to draw specific forms implies additional recruitment of attributes concerning their spatial dimensions and meaning. The responses in the angular gyrus were limb-independent which would imply that putative intention precedes effector specification. This may include accompanying 'pre-articulation' of silent fast word repeat in the two tasks. An argument against the explanation that the angular gyrus activation only represented silent speech is that a similar response profile would be expected in Broca's area, which was not the case.

### Temporal cortex in writing

We identified two (posterior) temporal cortex regions involved in writing and drawing. Bilateral activation of the iTG was present during drawing with either hand as well as left-hand writing, while its activation was only left-sided during right-hand writing. The mTG activation, extending into the superior temporal sulcus, was exclusively left-sided and particularly seen during right-hand writing while, at relaxed threshold, also during left-hand writing. This common left-hemisphere lateralization suggests a writing-related coherence between the left mTG and iTG activations. In writing, the left posterior iTG has been proposed to play a role in retrieving stored representations of written word forms or grapheme images [40,46]. Such form representation implies a vision-related modality which is indeed consistent with the interactions between this inferior temporal region and the fusiform gyrus, a key structure in visual processing of object form and face perception in both hemispheres [77,78]. In the left hemisphere, the mid-fusiform region makes a strong contribution to visual word processing [79–81] with a regional differentiation in selectivity for levels of letter-word complexity [82], while it is functionally interconnected with the posterior iTG concerning such early word recognition [83]. In further bottom-up processing of these orthographical elements in reading, interactions with the posterior mTG (superior temporal sulcus) have been proposed to specifically support orthographical to phonological conversion [83–86]. The left anterior sTG activation was most pronounced during writing with both hands and it was least during tapping, suggesting a gradually increasing semantic demand.

In our study, auditory-presented instructions specified the performance of writing and drawing without the option to read the written text or to look at the results of drawing. The posterior iTG activations in these two conditions thus point at top-down neuronal processing recruiting vision-related form elements by audition. This is consistent with attentional enhancement of this region when using visual stimuli [87]. Although activation in the left iTG was stronger during writing than drawing, its involvement in both tasks emphasizes that the brain classifies orthographical elements and basic forms such as circles and triangles in a similar way. The exclusively writing-related activation of the left posterior mTG, on the other hand, is consistent with its role in the conversion of phonological to orthographical elements [88,89]. This specific processing step in dictated writing thus seems to be additional to the general transition from an auditory to a vision-related modality. In our experiment, this role of the mTG in phonological-orthographical conversion was possibly identified because its involvement in a wider spectrum of semantic processing [88–90] was similarly present in the semantic content of the spoken text that specified which figure had to be drawn.

### Cerebellar and basal ganglia contributions to writing

The coherent activations in the anterior and posterior cerebellum we found in this study have been described earlier [38,42,45]. In this, the anterior lobe activation represents its contribution to the basic motor function underlying ipsilateral hand movement. This ipsilateral relationship reflects the cerebellar role in supporting the interconnected motor cortex of the contralateral hemisphere [91]. Similarly, the posterior cerebellar lobe is interconnected with contralateral cortical regions implicated in cognitive functions [91–93]. The right-sided dominance of the posterior cerebellar activation in writing, irrespective of the hand used for writing, can thus be explained by its contribution to the dominant left-hemisphere function underlying (written) language [45].

We did not see a limb-independent striatal contribution to writing. On the other hand, while all tasks recruited posterior putamen activation contralateral to the hand of action, the anterior putamen was exclusively activated during the higher-order motor tasks writing and drawing, only in the left hemisphere and only when performed with the right hand. This lateralized contribution to the organization of particularly complex hand movements suggest that the left anterior putamen represents a specific node in left-hemisphere circuitry that characterizes right-hand dominance. The putamen segregation between simple and complex movements would fit a general organization of parallel cortico-basal ganglia loops described for motor and non-motor functions [94]. The left thalamic activations that were seen not only in right- but also in left-hand writing, contrasted to tapping, points at an aspect of writing beyond movement characteristics [95–97]. Such left thalamic function is anatomically consistent with its position as an outflow target of the right cerebellum and would thus functionally mediate non-motor functions of right posterior cerebellar lobe as described in the previous paragraph.

### An integrated writing network

In the first part of this discussion we treated the logic of parallel processing streams, particularly based on a perspective of higher-order motor control, which was followed by discussing functions that have been described for distinct cortical regions identified in the present study. This provides arguments to further specify coherence between the putative processing streams described above. We conclude that the auditory specifications for writing and drawing evoke orthographical and visual figure form information, respectively. This is inferred from the involvement of the posterior iTG in conditions without visual stimuli. It is in this vision-related format that perceptual information is brought to the level of sensorimotor transformations organized in a dorsal parietal-premotor pathway. Such temporal-parietal interaction matches the general coherence between ventral and dorsal visual pathways, thus facilitating the use of specific form and object features as landmarks of environmental space within which purposeful action is performed [98]. In this, we saw that apparently orthographical and figure form information is similarly treated. Such similarity supports the view that orthographical information is processed in the superior parietal cortex without additional semantic information, which is consistent with the superior parietal responses Brownsett and Wise observed only during writing and not during speech [51].

While the posterior mTG plays a crucial role in phonological-orthographical conversion, fuelling the posterior iTG, we further argued that the strong similarity in response profiles of this region and Broca's area reflected its contribution to semantic processing in writing by dictation, which is indeed consistent with its general contribution to this linguistic aspect [90,99]. In this way, our results support and further specify a dual-route model concerning semantics and phonological elements implicated in auditory-motor transformations in language [88,100,101]. A difference with these previously proposed models, which particularly concerned speech, is that we distinguish a non-semantic inferior temporal—superior parietal pathway from a semantic perisylvian processing loop comprising the left posterior mTG and Broca's area / left PMv. A final stage of integration between orthographical and semantic elements may be achieved by interactions between the left PMd, Broca's area and the PMv. With regard to the premotor cortex, the antero-inferior portions of both the left PMd and PMv were writing specific, relative to drawing.

### Limitations of the study

The writing and drawing tasks were designed in such a way that they were considered balanced for basic sensorimotor parameters. This was achieved by maintaining similar 10 s timeframes of either cursively writing 3 to 4 words or drawing series of 3 to 4 figures. A single cursively written word was thus regarded to be the performance equivalent of a single figure. Moreover, as instructions were given aurally, 3 to 4 words similarly constituted the phonetic units for a sentence or a figure series. Indeed, the words were neither spelled, nor written in blocked letters. The absence of activation in the primary sensory and motor cortices provided support for the balance aimed at. Alternatively, one might argue that a single letter would be the best writing equivalent of a figure. In our study, this would imply that more letters than figures were used, possibly introducing inappropriately balanced conditions. To make a design with appropriately balanced letters and figures, both at the level of performance and complexity of instructions, would imply a different study with questions complementary to, but beyond the present study.

We did not obtain quantitative behavioral measures in our study. In this respect, one cannot exclude the possibility that differences in performance had an effect on the results, for example associated with differences in accuracy between the right- and left-hand tasks. On the other hand, the absence of primary sensorimotor activation in the performed comparisons provided support for the conclusion that activations were related to the higher-order aspects of these tasks.

To conclude, writing by dictation without feedback from reading makes use of an initial phonological—orthographical conversion in the left temporal lobe. This enables the left posterior iTG to offer 'vision-related' information to dorsal parietal-premotor circuitry for subsequent sensorimotor transformation. One may even speculate that in the natural circumstance of writing, this ventral temporal involvement serves anticipated reading during the evolving written text. The cerebral organization underlying this sensorimotor transformation of non-semantic elements is similarly implicated in drawing figure forms specified by audition. The left posterior mTG plays a central role in dividing the non-semantic and semantic elements in the dictated text, of which the semantic information is transferred along a putative perisylvian loop to gain access to Broca's area and the left anterior PMv. Final integration between these ventral frontal regions and the left PMd highlights the antero-inferior segment of the latter as a core writing center in the brain, historically coined Exner's area.
